# Mixed-Dimensional Assembly Strategy to Construct Reduced Graphene Oxide/Carbon Foams Heterostructures for Microwave Absorption, Anti-Corrosion and Thermal Insulation

**DOI:** 10.1007/s40820-024-01447-9

**Published:** 2024-06-17

**Authors:** Beibei Zhan, Yunpeng Qu, Xiaosi Qi, Junfei Ding, Jiao-jing Shao, Xiu Gong, Jing-Liang Yang, Yanli Chen, Qiong Peng, Wei Zhong, Hualiang Lv

**Affiliations:** 1 College of Physics, Guizhou Province Key Laboratory for Photoelectrics Technology and Application, Guizhou University, Guiyang City, 550025 People’s Republic of China; 2 College of Materials and Metallurgy, Guizhou University, Guiyang City, 550025 People’s Republic of China; 3 National Laboratory of Solid State Microstructures and Jiangsu Provincial Laboratory for NanoTechnology, Nanjing University, Nanjing, 210093 People’s Republic of China; 4 Department of Materials Science and Laboratory of Advanced Materials, Fudan University, Shanghai, 200433 People’s Republic of China

**Keywords:** Multifunctionality, Reduced graphene oxide/carbon foams, 2D/3D van der Waals heterostructures, Electromagnetic wave absorption, Thermal insulation

## Abstract

Considering the serious electromagnetic wave (EMW) pollution problems and complex application condition, there is a pressing need to amalgamate multiple functionalities within a single substance. However, the effective integration of diverse functions into designed EMW absorption materials still faces the huge challenges. Herein, reduced graphene oxide/carbon foams (RGO/CFs) with two-dimensional/three-dimensional (2D/3D) van der Waals (vdWs) heterostructures were meticulously engineered and synthesized utilizing an efficient methodology involving freeze-drying, immersing absorption, secondary freeze-drying, followed by carbonization treatment. Thanks to their excellent linkage effect of amplified dielectric loss and optimized impedance matching, the designed 2D/3D RGO/CFs vdWs heterostructures demonstrated commendable EMW absorption performances, achieving a broad absorption bandwidth of 6.2 GHz and a reflection loss of − 50.58 dB with the low matching thicknesses. Furthermore, the obtained 2D/3D RGO/CFs vdWs heterostructures also displayed the significant radar stealth properties, good corrosion resistance performances as well as outstanding thermal insulation capabilities, displaying the great potential in complex and variable environments. Accordingly, this work not only demonstrated a straightforward method for fabricating 2D/3D vdWs heterostructures, but also outlined a powerful mixed-dimensional assembly strategy for engineering multifunctional foams for electromagnetic protection, aerospace and other complex conditions.

**Supplementary Information:**

The online version contains supplementary material available at 10.1007/s40820-024-01447-9.

## Highlights

- Reduced graphene oxide/carbon foams (RGO/CFs) vdWs heterostructures are efficiently fabricated via a simple mixed-dimensional assembly strategy.
- Linkage effect of optimized impedance matching and enhanced dielectric loss abilities endows the excellent microwave absorption performances of RGO/CFs vdWs heterostructures.
- Multiple functions such as good corrosion resistance performances and outstanding thermal insulation capabilities can be integrated into RGO/CFs vdWs heterostructures.

**Supplementary Information:**

The online version contains supplementary material available at 10.1007/s40820-024-01447-9.

## Graphical abstract

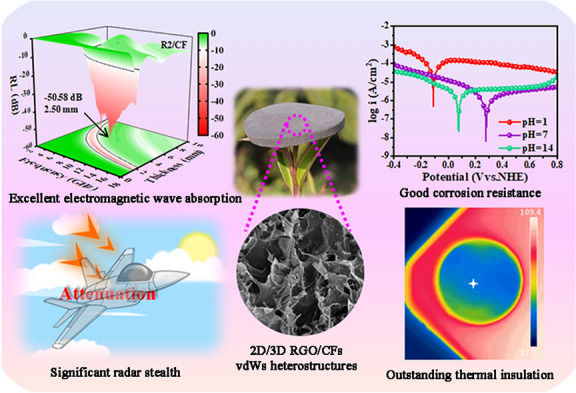

## Introduction

As the continuous and rapid progress of electronic communication technology, the popular intelligent electronic equipment brings convenience to people’s life. Meanwhile, it also hides serious electromagnetic (EM) pollution and threatens people’s health [1–3]. Consequently, the focus on designing outstanding materials and structures to effectively improve EM wave (EMW) absorption performances has increasingly intensified. According to the actual application requirements, the desired EMW absorption materials are appraised not only by the characteristics of “strong,” “broad,” “thin” and “lightweight,” but also by their high environmental adaptability such as good anti-corrosion and superior thermal stability [4]. Accordingly, biomass-derived [5] or chemically synthesized carbon-based materials from zero dimension (0D) to three dimension (3D) such as carbon nanocages/microspheres [6, 7], carbon nanofibers (CNFs) [8], graphene (G) [9], and carbon aerogels [10] are deemed as the extremely attractive candidate substances for developing perfect EMW absorption materials relying with their extraordinary electrical conductivity, light quality, high physical/chemical stability, and so on [11, 12]. Unfortunately, the poor impedance matching characteristic and attenuation mechanism greatly hinder the improvement of EMW absorption performances [13]. In order to effectively solve these problems, different methods and strategies have been proposed. For examples, a new nano‑micro engineering was presented by Cao’s team, which could modulate the inner porous structure of NiCo_2_O_4_ nanofibers and further effectively regulated the EMW absorption performances by boosting its charge transport capacity. More importantly, this simple strategy for constructing diverse microstructures could be extended to other EM functional materials [14]. Che and co-workers reported a pioneering galvanic engineering for constructing core@shell structure nanohybrids to exploit efficient EMW absorbers. Wondrously, the diversity of heterogeneous nanoparticle shell composition composed of single-metal or bimetallic was controlled and quantitatively regulated through this general programmable strategy [15]. Recently, Ji et al. employed phase engineering strategy to boost dielectric loss through regulating amorphous/crystalline heterophase of γ-Fe_2_O_3_ nanosheets. Concluding from the results, compared with the pure amorphous and bare crystalline, the designed composites exhibited an effective absorption bandwidth (EAB), which was attributed to heterointerface provided by different phase structures [16]. Similarly, Reza Peymanfar et al. and Zhang’s team successfully promoted the EMW absorption performances of MgFe_2_O_4_-based materials and NbS_2_ through manipulating the phase and morphology, respectively [17, 18]. Additionally, Wu’s group proposed a vacancy engineering of Se-doped CoS_2_ and S-doped CoSe_2_ through an anion-doping. Benefitting from much superiority of improved electronic conductivity and numerous polarization centers caused by vacancy sulfur and selenium, the EMW absorption performances were successfully optimized [19]. In general, the previously reported results revealed that EMW absorption performances were significantly boosted through the meticulous regulation of morphology and microstructures, phase and components, defect and interfacial effects.

Mixed-dimensional heterostructures, especially van der Waals (vdWs) heterostructures, are undoubtedly desirable structures for constructing high-performance EMW absorption materials by virtue of large specific surface area, abundant interfaces, multi-dimensional components, and so on [20, 21]. For instance, Pan and his colleagues synthesized multi-dimensional heterostructures, which were composed of 3D carbon nanocoils, two-dimensional (2D) graphene, one-dimensional (1D) CNFs and 0D nanoparticles. According to the results, the impedance matching and EMW absorption characteristics could be regulated by modifying the growth parameters of CNFs and nanoparticles [22]. Liu’s group designed and constructed multi-dimensional hybridized structures of 3D N-doped carbon aerogels with attachment of 0D Ni/MnO nanoparticles. In consequence, compared with pure 3D N-doped carbon aerogels, in situ incorporation of 0D Ni/MnO particles greatly adjusted the absorption capacity and achieved a ultrawide absorption bandwidth [23]. Recently, Wu et al. constructed 0D selenide nanoparticles@2D carbon nanosheets@1D CNFs mixed-dimensional composites for multi-functional applications. With respect to the extraordinary EMW absorption performances of composites, it was mainly ascribed to the synergistic effect combined with good conductive networks, abundant space gap and rich heterointerfaces [24]. Besides the strong absorption and wide bandwidth, perfect EMW absorbing materials with excellent stability and versatility to satisfy the ever-increasing demands in the changeable practical environment will be a key research direction in the future. However, effectively incorporating the multiple functionalities including EMW absorption capability, heat protection, and resistant to corrosion into carbon materials still faces huge challenges so far.

Considering the presented aspects, herein, 2D/3D reduced graphene oxide/carbon foams (RGO/CFs) vdWs heterostructures were meticulously engineered and synthesized via freeze-drying, immersing absorption and thermal treatment. The obtained results suggested that their unique structures and components induced the linkage effect of optimized impedance matching and enhanced dielectric loss abilities, leading to the significant EMW absorption, good anti-corrosion as well as thermal insulation performances of 2D/3D RGO/CFs vdWs heterostructures. Accordingly, our works not only demonstrated an efficient pathway to produce 2D/3D RGO/CFs vdWs heterostructures, but also provided a facile mixed-dimensional assembly strategy to develop multifunctional carbon materials for the great potential in complex and variable environments.

## Experimental Section

### Fabrication of 3D Cellular Chitosan/g-C_3_N_4_ Foams (CGFs)

In a typical experiment, the 3D CGFs were prepared through a simply equipped freeze-drying process. Initially, yellow g-C_3_N_4_ powder as viscosity modifier was acquired by a thermal decomposition of urea. And g-C_3_N_4_ powder (60 mg) was ultrasonically dispersed into deionized water (60 mL) for 30 min to prepare the g-C_3_N_4_ aqueous dispersion. After that, chitosan powder (2.4 g) was completely dispersed into the above dispersion. Subsequently, glacial acetic acid (1.2 mL) was injected into the chitosan/g-C_3_N_4_ aqueous dispersion under magnetic stirring to synthesize the yellow chitosan/g-C_3_N_4_ hydrogel precursor. Then, each 13 g of chitosan/g-C_3_N_4_ hydrogel was transferred into glass garden and placed at room temperature until the bubble disappeared. After frozen at ca. − 60 °C, the ice templates were removed after the vacuum freeze-drying treatment to obtain 3D cellular CGFs.

### Fabrication of 2D/3D GO/CGFs and RGO/CFs vdWs Heterostructures

Firstly, few-layer GO could be synthesized using the previously reported route [25]. Particularly, the yellow CGFs were placed in the oven at 80 °C for 48 h to further promote the cross-linking reaction. Afterward, deionized water was employed to clean the CGFs for removing the residual glacial acetic acid. The obtained wet CGFs should be squeezed as much as possible to remove excess deionized water. At the same time, GO aqueous dispersions with different concentrates (2, 4, and 6 mg mL^−1^) were obtained by ultrasonic dispersing different amounts of GO (40, 80, and 120 mg) in 20 mL deionized water for 30 min, respectively. Next, the extruded CGFs were separately immersed into different concentrations of GO dispersions under stirring until they were saturated, which were subsequently placed into a freeze-dryer to produce GO/CGFs vdWs heterostructures. For easy description, the obtained GO/CGFs with different contents of GO were named as G2/CGF, G4/CGF and G6/CGF, respectively. Finally, the lyophilized GO/CGFs heterostructures were carbonized at 650 °C (model BTF-1200C, Anhui BEQ Equipment Technology Co, Ltd.) for 2 h in Ar to obtain the corresponding RGO/CFs vdWs heterostructures, which were denoted as R2/CF, R4/CF and R6/CF, respectively. For comparison, the 3D cellular CFs without 2D RGO nanosheets attachment were obtained through directly carbonization process of CGFs. Aiming at deeply exploring the influence of carbonization temperature, taking G2/CGF as a research object, the carbonization process was also carried out under 600 and 700 °C to produce the corresponding RGO/CFs heterostructures (named as R2/CF-600 and R2/CF-700).

### Characterization

For making sure the phases, morphology, elements mapping and compositions of samples, emission scanning electron microscopy (FE-SEM), energy dispersive spectrometer (EDS), Fourier transform infrared (FTIR) spectrum, Raman spectra, X-ray photoelectron spectrometer (XPS) and X-ray powder diffractometer (XRD) were successively carried out. To investigate EMW absorption properties, the obtained specimens (15, 20, and 25 wt%) were mixed with paraffin to compress into a series of toroidal shapes (3.0 mm inner diameter and 7.0 mm outer diameter). A vector network analyzer was used to measure their EM parameters using the coaxial-line method from 2.0 to 18.0 GHz.

## Results and Discussion

### Composition, Microstructure, EM and EMW Absorption Properties of RGO/CFs vdWs Heterostructures by Regulating Content of GO

**Fig. 1:**
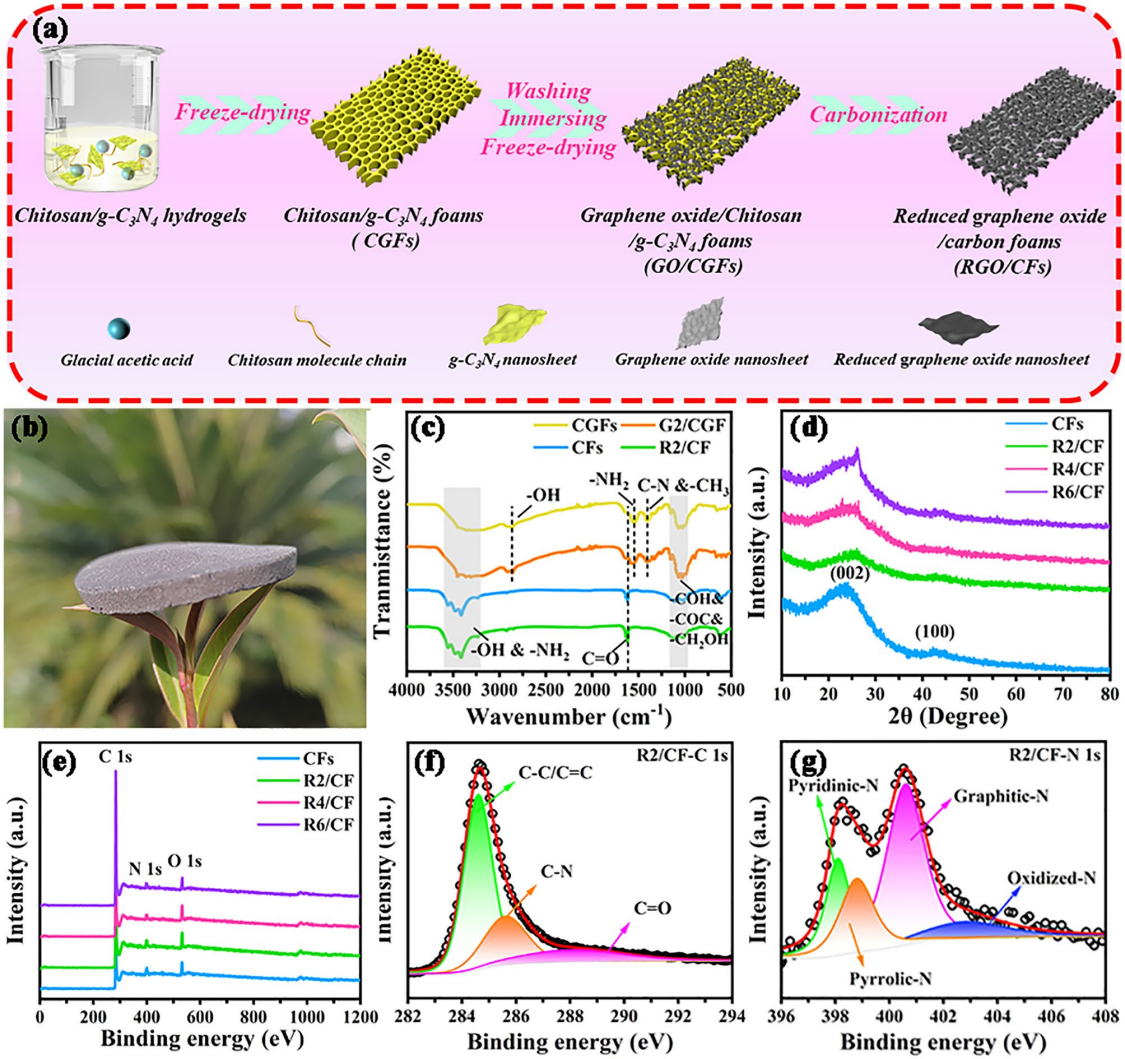
**a** Experimental diagram of 2D/3D GO/CGFs and RGO/CFs vdWs heterostructures, **b** digital image of R2/CF standing on leaves, **c** FTIR spectra, **d** XRD patterns, **e** XPS spectra of CFs and RGO/CFs. **f, g** C 1*s* and N 1*s* XPS spectra of R2/CF

As schematically depicted in Fig. 1a, 2D/3D RGO/CFs vdWs heterostructures can be efficiently fabricated in a large scale through a simple consecutive three steps: (i) obtaining CGFs by freeze-drying technique, (ii) preparation of GO/CGFs through immersing absorption and secondary freeze-drying process, and (iii) the finial formation of 2D/3D RGO/CFs vdWs heterostructures via the subsequent carbonization processes. Like the previous low-density EMW absorbers [26], RGO/CFs vdWs heterostructures have a very low density of ca. 45 mg cm^−3^, which are calculated by the dimension of 35 mm diameter and 3 mm height. As shown in Fig. 1b, taking R2/CF sample as an example, R2/CF can be easily supported by a leaf without any alteration of its external form, confirming the ultra-lightweight features of RGO/CFs heterostructures. Figure 1c shows the FIIR spectra of CGFs, G2/CGF, CFs, and R2/CF. The analysis of FIIR curves for CGFs and G2/CGF reveals that the –OH peaks undergo an evident red shift from ca. 3430 to ca. 2900 cm^−1^, which are primarily ascribed to the appearance of hydrogen bonds caused by the superfluous glacial acetic acid [27]. On account of their similar oxygen-containing functional groups of GO and chitosan, CGFs and G2/CGF samples display the similar FIIR curves, showing the characteristic peaks of hydrophilic groups. Compared to CGFs and G2/CGF, the FIIR results for CFs and R2/CF samples reveal that these characteristic peaks of –COH, –COC and –CH_2_OH (within 1000–1200 cm^−1^) are diminished, and the –NH_2_ and C–N peak intensities are significantly disappeared, which implies the reduction of hydrophilic groups in chitosan and GO during the pyrolysis process [23]. Furthermore, the C=O peak is still pronounced from the obtained CFs and R2/CF, which is beneficial to induce polarizations for the attenuation of EMW. As provided in Fig. 1d, the obtained CFs and RGO/CFs show the broad peaks of graphitic carbon at 24° and 44°, respectively [28]. Specially, the disappearance of diffraction peak corresponding to GO in XRD pattern suggests the successful reduction of GO, which is consistent with the analysis of FTIR. With reference to the previous reports, no obvious diffraction peak of g-C_3_N_4_ (27°) appears, demonstrating complete decomposition of few g-C_3_N_4_ after pyrolysis [29]. To further investigate the surface chemistry of samples, the XPS measurement was conducted. The XPS survey spectrums of CFs and RGO/CFs exhibit O 1*s*, N 1*s*, and C 1*s* characteristic peaks in Fig. 1e, providing strong evidence of N-doping. In the C 1*s* orbit of R2/CF (Fig. 1f), the spectrum is deconvoluted as a combination of three characteristic peaks: 288.0, 285.6, and 284.6 eV, which correspond to C=O, C–N, and C–C/C=C [30]. In marked in Fig. 1g, the N 1*s* XPS spectrum of R2/CF sample is presented to further confirm the bonding configuration of N, which indicate the presences of oxidized-N, graphitic-N, pyrrolic-N and pyridinic-N, respectively [31]. Additionally, the comparison of the high-resolution spectra C 1*s* and N 1*s* for R4/CF (Fig. S1a, b) and R6/CF (Fig. S1c, d) suggests the similar composite components. It is well known that pyrrolic-N and pyridinic-N as polarization centers and graphitic-N as conduction loss enhancer help to improve the dissipation of EMW [32].

**Fig. 2:**
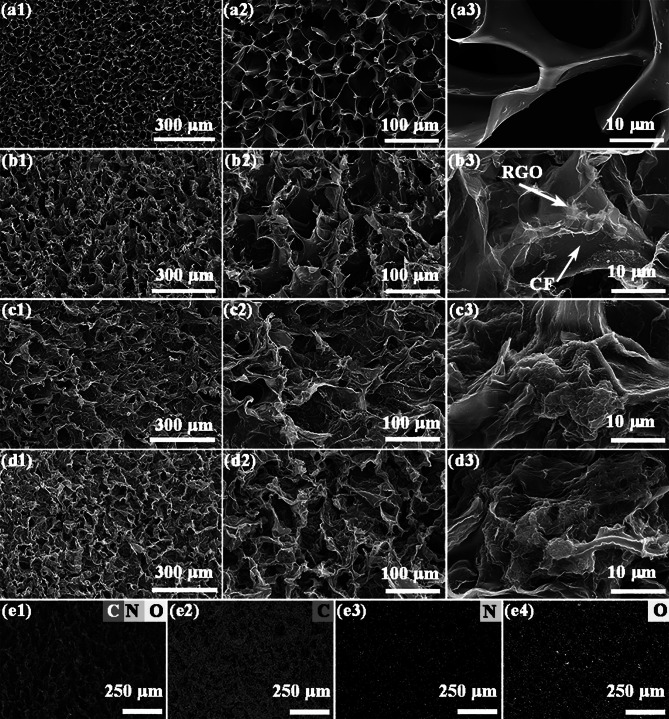
**a1**–**d3** FE-SEM images of CFs, R2/CF, R4/CF and R6/CF, **e1–e4** EDS element mapping images of R2/CF, respectively

To further study their structures, the precursor CGFs and GO were characterized by FE-SEM and TEM in Fig. S2. Before thermal treatment, CGFs exhibit coarse skeleton and small pores (Fig. S2a, b) and GO shows typical 2D tulle-like nanosheets (Fig. S2c, d). After processing, the as-prepared CFs and RGO/CFs were also investigated by FE-SEM. From Fig. 2a1–a3, the obtained CFs sample manifests a uniform faveolate configuration with a comparatively smooth surface. The generation of the dense channels can be attributed to the formation of ice crystal and subsequent sublimation under the treatment of freeze-drying. Compared with CFs, the FE-SEM observations from Fig. 2b1–b3 demonstrate that the channels are filled with RGO nanosheets in large scale and 2D RGO nanosheets are firmly affixed to the 3D skeleton surface of CFs via the van der Waals forces, constructing a typical 2D/3D vdWs heterostructures and generating large quantities of solid–void interfaces. To further test this idea, FE-SEM images of R4-CF were gained and dipicted in Fig. 2c1–c3. The investigations reveal that the R4/CF exhibits much rougher frameworks and denser channels than the R2/CF sample due to the attachment of much more RGO. And the R4/CF sample is also the representative 2D/3D vdWs heterostructures, which consists of 2D RGO nanosheets and 3D CFs. With a further increase in the GO content, the SEM observations reveal that the channel structure of R6/CF becomes more blurred, and RGO nanosheets clearly stack into clusters and evidently accumulate on the surface of skeleton (Fig. 2d1–d3). To further determine the elemental distribution, EDS elemental mapping images of R2/CF are provided in Fig. 2e1–e4. The results illustrate that the elements of O, N, and C are evenly distributed throughout the R2/CF sample, which is consistent with the XPS analysis. Overall, the acquired outcomes demonstrate that RGO/CFs 2D/3D vdWs heterostructures can be fabricated simply and efficiently through our proposed route. By adjusting the initial concentration of GO, the RGO content and morphology of designed RGO/CFs can be effectively manipulated. More importantly, the obtained 2D/3D RGO/CFs vdWs heterostructures build the good conductive networks and provide abundant interfaces of void–solid, which promote the multiple scattering, reflections and attenuation of EMW [33].

**Fig. 3:**
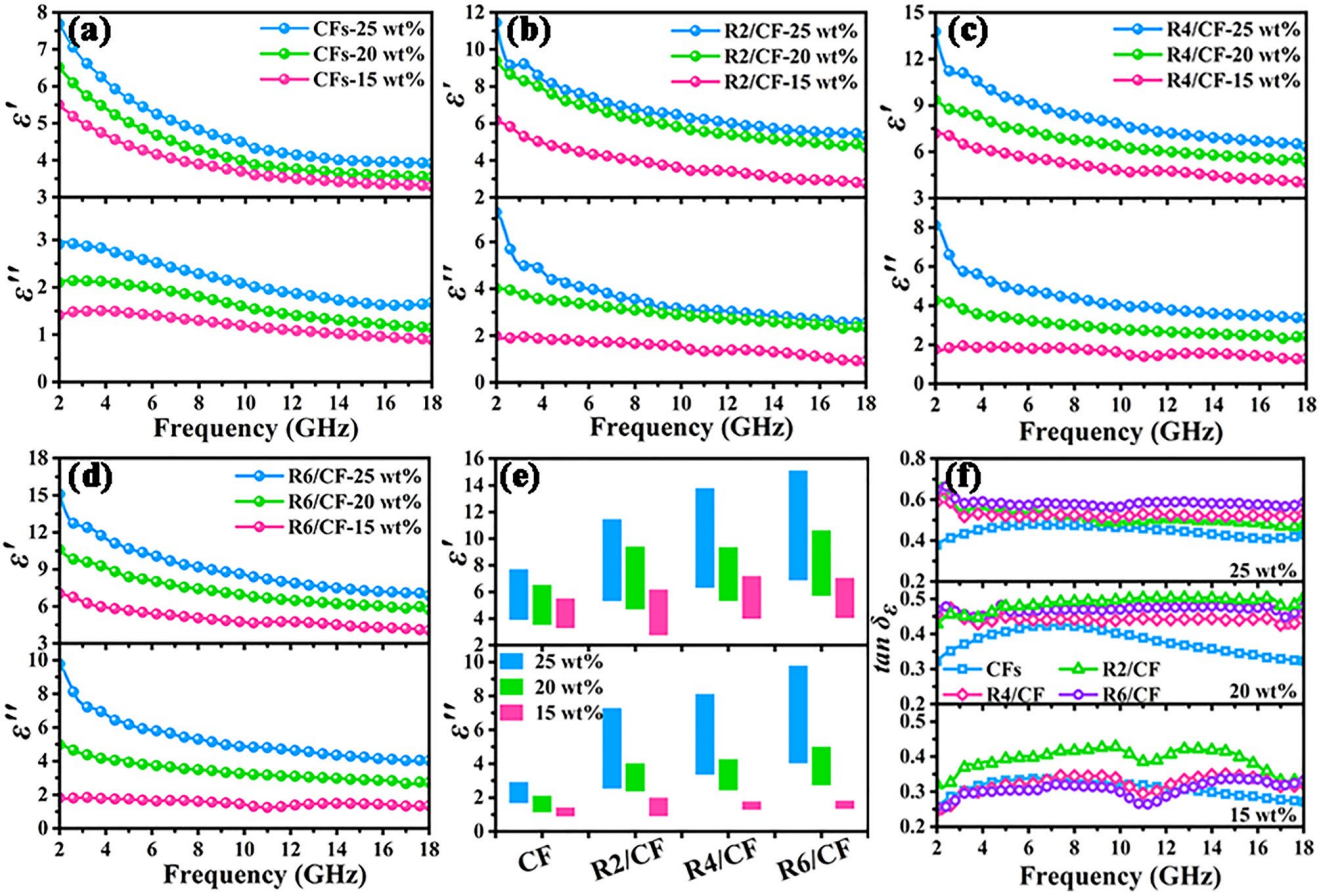
**a–d** $\varepsilon^{\prime}$ and $\varepsilon^{\prime\prime}$ values, **e** comparison $\varepsilon^{\prime}$ and $\varepsilon^{\prime\prime}$ values, **f** dielectric loss tangent values for CFs and RGO/CFs with different filling ratios

For the sake of confirming the aforementioned analyses, Fig. 3 offers the EM parameters and dielectric loss tangent ($\tan \delta_{E} = \frac{{\varepsilon^{\prime\prime}}}{{\varepsilon^{\prime}}}$) for obtained CFs, R2/CF, R4/CF and R6/CF with the packing ratios of 15, 20, and 25 wt%. Due to the non-magnetic characterization of RGO and carbon [34], the $\varepsilon^{\prime}$ and $\varepsilon^{\prime\prime}$ values determine EMW absorption characteristics of designed absorbers, which are related to storage and dissipation capacity, respectively [35]. Intuitively, as presented in Fig. 3a-d, all the samples exhibit the degraded $\varepsilon^{\prime}$ and $\varepsilon^{\prime\prime}$ values within the tested frequency range, which is in line with the frequency dispersion phenomenon of carbon materials [36]. Specifically, the $\varepsilon^{\prime}$ and $\varepsilon^{\prime\prime}$ values (Fig. 3a) for 3D cellular CFs with a filling ratio of 15 wt% are relatively small, which decrease from 5.501 to 3.289, and 1.411 to 0.892, respectively. With the increasing of filler loading, the $\varepsilon^{\prime}$ and $\varepsilon^{\prime\prime}$ values of CFs with the filling ratios of 20 and 25 wt% range from 6.519 to 3.535 and 2.098–1.142, 7.688–3.915 and 2.907–1.676, respectively. Similar to the previous findings [37], the relatively low values of complex permittivity for 3D CFs sample should be ascribed to the insufficient filling amount, which makes it difficult to form a complete conductive network. The $\varepsilon^{\prime}$ and $\varepsilon^{\prime\prime}$ values (Fig. 3b) of R2/CF with different filling ratios are as follows: 6.162–2.753 and 1.984–0.907, 9.385–4.695 and 4.019–2.370, 11.449–5.319 and 7.268–2.540. And the designed R2/CF sample presents much higher values of $\varepsilon^{\prime}$ and $\varepsilon^{\prime\prime}$ than 3D CFs with a same filling ratio, demonstrating the improved ability to store and attenuate EMW energy. These situations are attributed to the attachment of 2D RGO nanosheets on 3D CFs contributing to construct the mix-dimensional vdWs heterostructures and form a dense conductive network, which accelerates electrons migration and hopping process [38, 39]. To further verify the above deduction, Fig. 3c, d presents the values of complex permittivity for R4/CF and R6/CF samples with the different filling ratios. As speculated, $\varepsilon^{\prime}$ and $\varepsilon^{\prime\prime}$ values of R6/CF are still significantly higher than that of R4/CF at the same filling ratios, which is mainly due to the gradual stacking of the RGO flakes together and the further reduction of the pore size, resulting in higher electrical conductivity. The visualized comparison $\varepsilon^{\prime}$ and $\varepsilon^{\prime\prime}$ values (as shown in Fig. 3e) of CFs, R2/CF, R4/CF and R6/CF samples further confirm the obtained analyses and SEM results, suggesting the effective modulation of EM parameters after incorporation of RGO. And their dielectric loss tangent values also indicate that the CFs and RGO/CFs present the steadily upward trend when the filling ratio increases from 15 to 25 wt% (as depicted in Fig. 3f), implying their improved dielectric loss capacities [40]. Furthermore, the comparative outcomes also manifest that the 2D/3D RGO/CFs vdWs heterostructures exhibit the superior dielectric loss abilities compared to CFs.

**Fig. 4:**
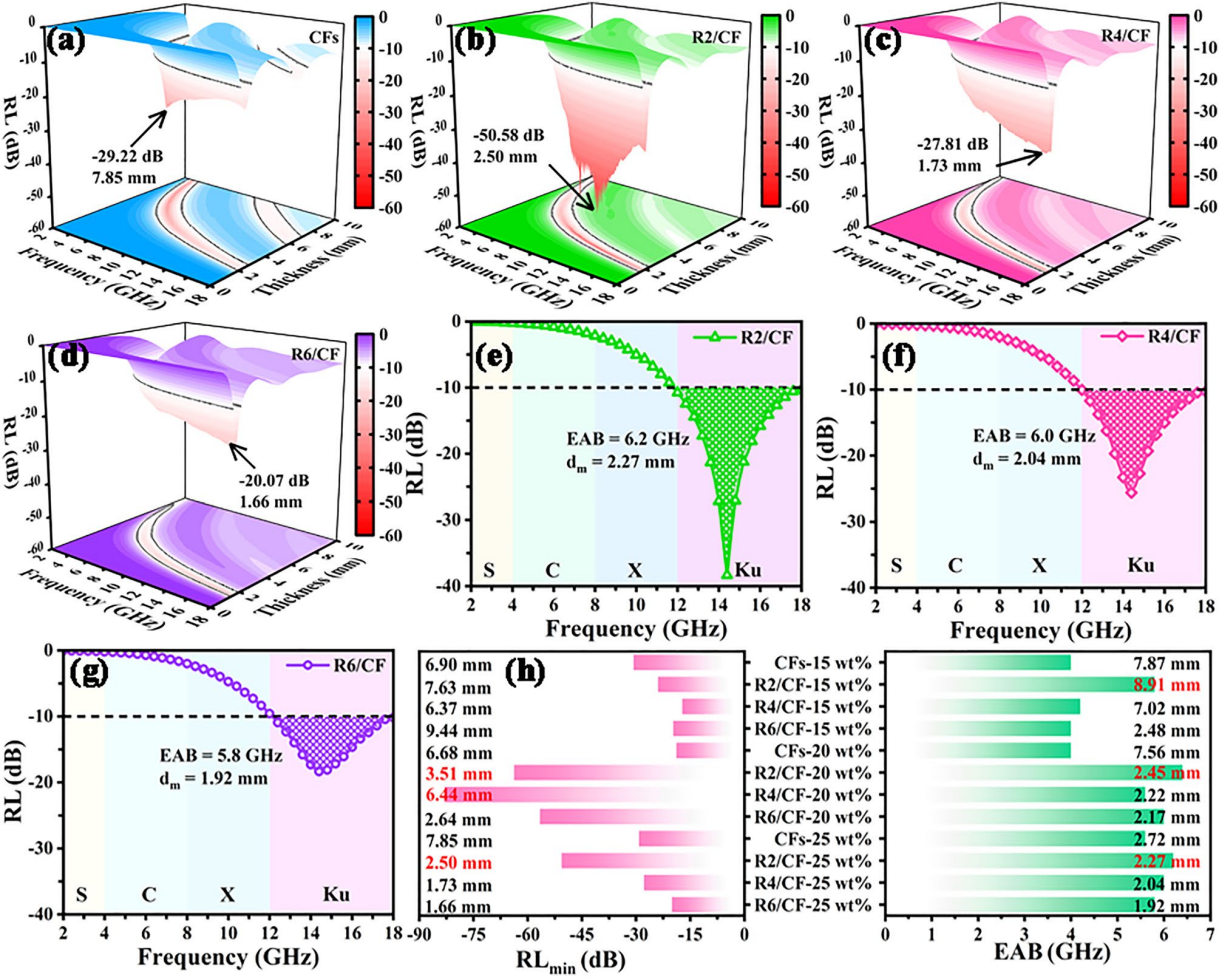
**a–d** 3D RL color maps, **e–g** EAB and d_m_ curves for R2/CF, R4/CF and R6/CF with a 25 wt% filling ratio. **h** Summarized RL_min_, EAB and d_m_ values for CFs, R2/CF, R4/CF and R6/CF with different filling ratios (15 wt%, 20 wt%, 25 wt%)

To study their EMW absorption performances, the reflection loss (RL) values were acquired on basis of transmission line theory and corresponding equations (Eq. S1) and (Eq. S2) in Supporting Information [41, 42]. As illustrated in Fig. 4a-d, the 3D RL color maps reveal that the *RL*_min_ values of CFs, R2/CF, R4/CF, and R6/CF samples with the filling ratio of 25 wt% are − 29.22, − 50.28, − 27.81, and − 20.07 dB. Their corresponding frequency locations and matching thicknesses (*d*_*m*_) values are 4.0 GHz and 7.85 mm, 12.8 GHz and 2.50 mm, 17.4 GHz and 1.73 mm, 17.2 GHz and 1.66 mm, respectively. Furthermore, the obtained CFs (Fig. S3), R2/CF (Fig. 4e), R4/CF (Fig. 4f) and R6/CF (Fig. 4g) samples display the EAB values of 5.6, 6.2, 6.0, and 5.8 GHz. And the corresponding *d*_m_ values are 2.72, 2.27, 2.04, and 1.92 mm, respectively. For comparison, Fig. S4 demonstrates the EM parameters and absorption performances of the pure GO. Without doubt, the extremely low EM parameters result in very poor performance. What is more, the attenuation constant ($\alpha$) curves for CFs and RGO/CFs calculated based on Eq. S3 are showed in Fig. S5. The gradually rising $\alpha$ values of RGO/CFs indicate the enhancement of EMW attenuation abilities compared to the CFs. More intuitively, Fig. 4h provides the comparison values of *RL*_min_, EAB and corresponding *d*_*m*_ for CFs, R2/CF, R4/CF, and R6/CF samples, which further confirm the tunable EM and EMW absorption properties of 2D/3D RGO/CFs vdWs heterostructures by regulating the introduction of RGO. Additionally, the comparison results (as presented in Fig. S6) between CFs and R2/CF based on quarter-wavelength matching theory were carried out. From that, all dots corresponding to the thickness-frequency nearly locate on the simulated curve, indicating the good coincidence between theoretical and experimental outcomes [43]. Consequently, the designed R2/CF sample exhibits the strong absorption capabilities, broad EAB as well as small matching thicknesses, which is a super-duper novel EMW absorbers.

### Impact of Thermal Treatment Temperature

**Fig. 5:**
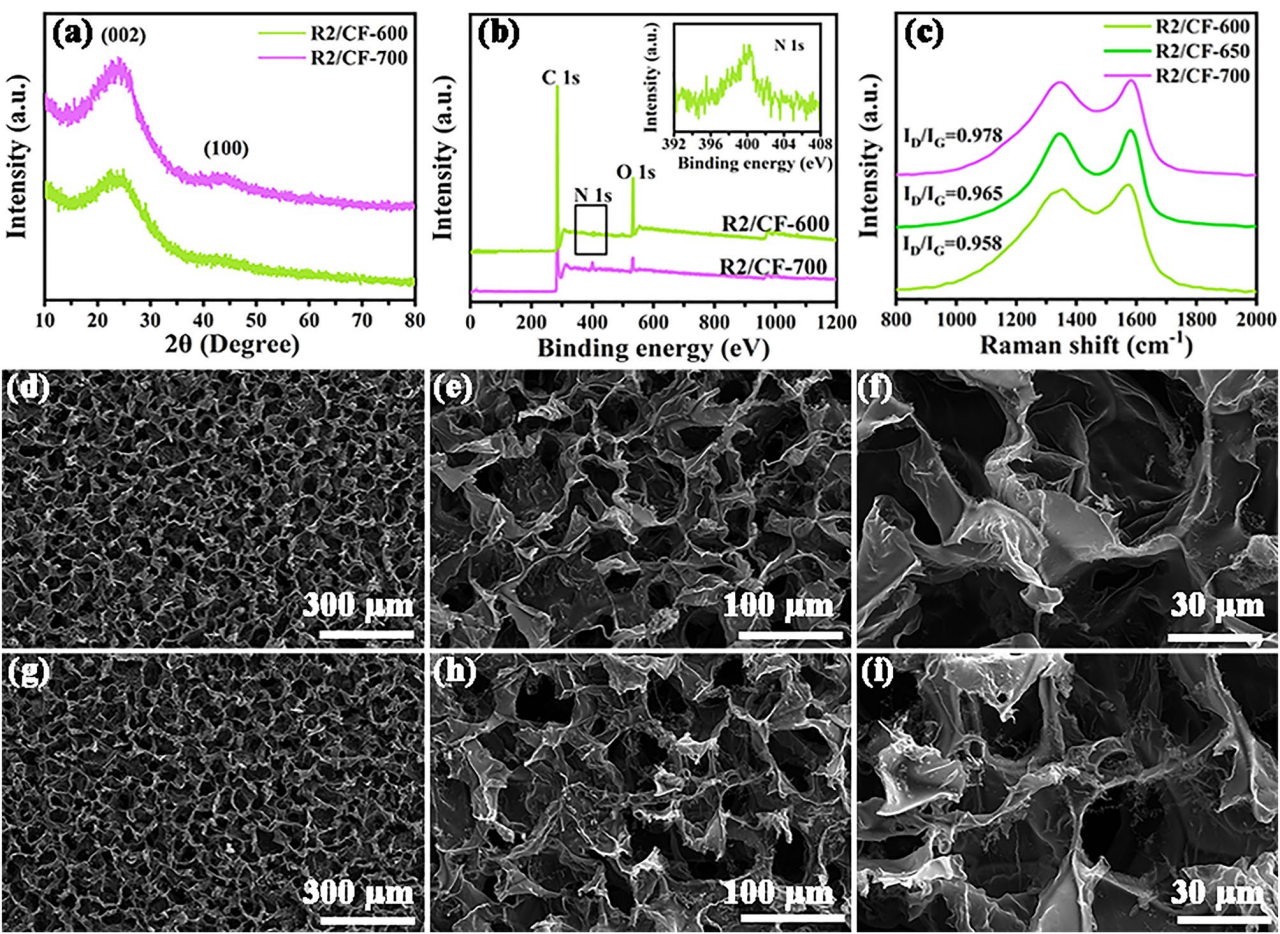
**a–c** XRD, XPS and Raman spectra for R2/CF-600, R2/CF-650 and R2/CF-700 samples, and SEM images for **d–f** R2/CF-600 and **g-i** R2/CF-700

To investigate the influence of thermal treatment temperature, the compositions, morphologies and EMW absorption characteristics of obtained R2/CF-600 and R2/CF-700 were deeply investigated. From Fig. 5a, the analysis of XRD reveals that the as-prepared R2/CF-600 and R2/CF-700 display the alike diffraction peaks, which are similar to the obtained R2/CF. Furthermore, the RGO/CFs vdWs heterostructures exhibit the increasingly XRD peak intensities corresponding to graphitic carbon with the carbonization temperature increasing from 600 to 700 °C, indicating the gradual increase in graphitic carbon content. As provided in Fig. 5b, the XPS analysis reveals the presence of N, O and C, which also distribute over the obtained R2/CF-600 and R2/CF-700 samples. Distinctly, the declining O 1*s* peak of the obtained samples suggests the reduced oxygen content with enhancing the thermal treatment temperature, which implies the improved reduction degree of G2/CGF. And in Fig. S7, C 1*s* and N 1*s* high-resolution spectra for R2/CF-600 and R2/CF-700 are provided. In particular, the strong C=O peak in Fig. S7a and the missing graphitic-N peak in Fig. S7b also suggest the low degree of carbonization at 600 °C. Conversely, the weakened C=O peak in Fig. S7c and the emergence of graphitic-N peak in Fig. S7d further confirm the deepening of reduction at 700 °C. In addition, Raman spectra are also provided. From Fig. 5c, all the obtained samples display two characteristic peaks at about 1345 and 1585 cm^−1^ corresponding to D and G band [44]. And their peak intensity ratios (*I*_D_/*I*_G_) are 0.958, 0.965, and 0.978. And the gradual increase in value coincides with the transformation from amorphous carbon to graphitic nanocrystals on basis of three-stage model [45, 46]. Thus, abundant C=C bonds in graphite nanocrystals generate a 2D plane and thus decrease the electrical resistivity [47]. One can see that the Raman spectra are accorded with the above-mentioned XRD and XPS outcomes. Same to R2/CF, the SEM investigations reveal that both the obtained R2/CF-600 (Fig. 5d-f) and R2/CF-700 (Fig. 5g-i) display the representative 2D/3D vdWs heterostructures in which 2D RGO nanosheets firm anchoring to 3D cellular structure, which implies that the influence of heat treatment temperature on the morphology can be ignored. In short, the content of graphitic carbon is modulated by regulating the heat treatment temperature, facilitating the optimization of their EM parameters and EMW absorption properties.

**Fig. 6:**
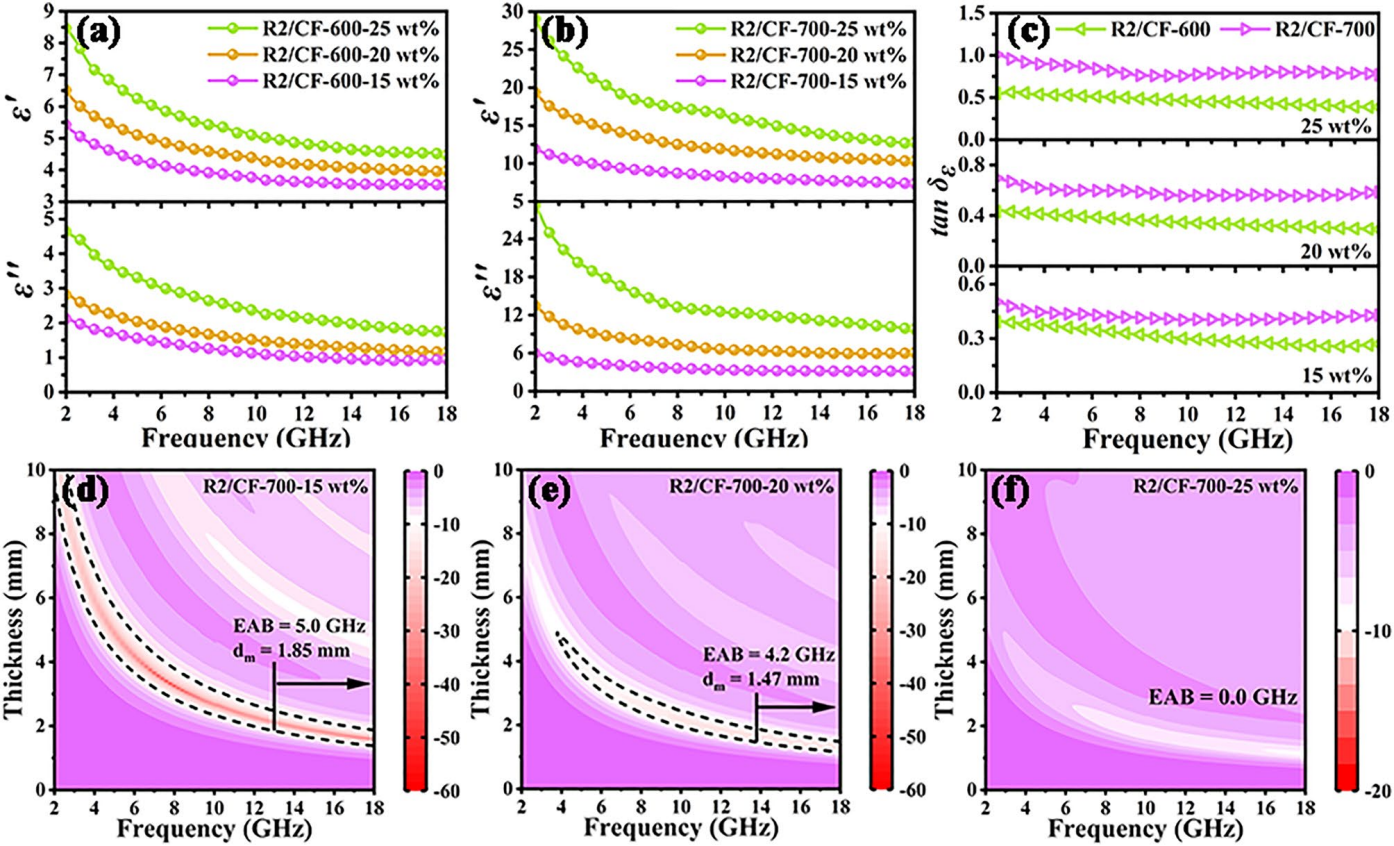
**a–c** $\varepsilon^{\prime}$, $\varepsilon^{\prime\prime}$ and $\tan \delta_{E}$ values for R2/CF-600 and R2/CF-700, and **d-f** 2D RL color maps for R2/CF-700 at the filling ratios of 15 wt%, 20 wt% and 25 wt%

To confirm the effect of carbonization temperature on their performance, EM parameters for R2/CF-600 and R2/CF-700 samples were also investigated. The achieved R2/CF-600 (Fig. 6a,) and R2/CF-700 (Fig. 6b) samples also present the gradually increasing values of $\varepsilon^{\prime}$ and $\varepsilon^{\prime\prime}$ when the filling ratio raises from 15 to 25 wt%. Furthermore, the outcomes reveal that RGO/CFs vdWs heterostructures at a same filler loading exhibit the evident enhancement in $\varepsilon^{\prime}$, $\varepsilon^{\prime\prime}$ and $\tan \delta_{E}$ values (as presented in Fig. 6c), which further confirms the adjustment of EM performances by the carbonization temperature. Additionally, the 2D RL map (as presented in Fig. 6d) suggests that the EAB and *RL*_min_ values for R2/CF-700 sample at 15 wt% are 5.0 GHz (from 13.0 to 18.0 GHz) and − 52.05 dB. And their corresponding *d*_m_ values are 1.85 mm and 3.32 mm at the frequency of 7.8 GHz, respectively. Equally, the obtained R2/CF-700 sample with a 20 wt% filling ratio (Fig. 6e) also displays the *RL*_min_ and EAB values of 14.80 dB and 4.2 GHz (13.8–18.0 GHz), and their matching thicknesses are 1.47 mm. And the too high complex permittivity (Fig. 6f) gives rise to impedance mismatching characteristic and poor EMW absorption properties of R2/CF-700 at a 25 wt% filling ratio [48]. Meanwhile, the other detailed EM parameters and absorption performances of both samples at different filling ratios are summarized in Table S1. According to the acquired outcomes, it is evident that the excellent EMW absorption performances of obtained 2D/3D RGO/CFs vdWs heterostructures are also tailored by modulating the thermal treatment temperature.

### Analyses on the Difference in EMW Absorption Properties, Radar Cross Section Simulation and Possible EMW Absorption Mechanism

**Fig. 7:**
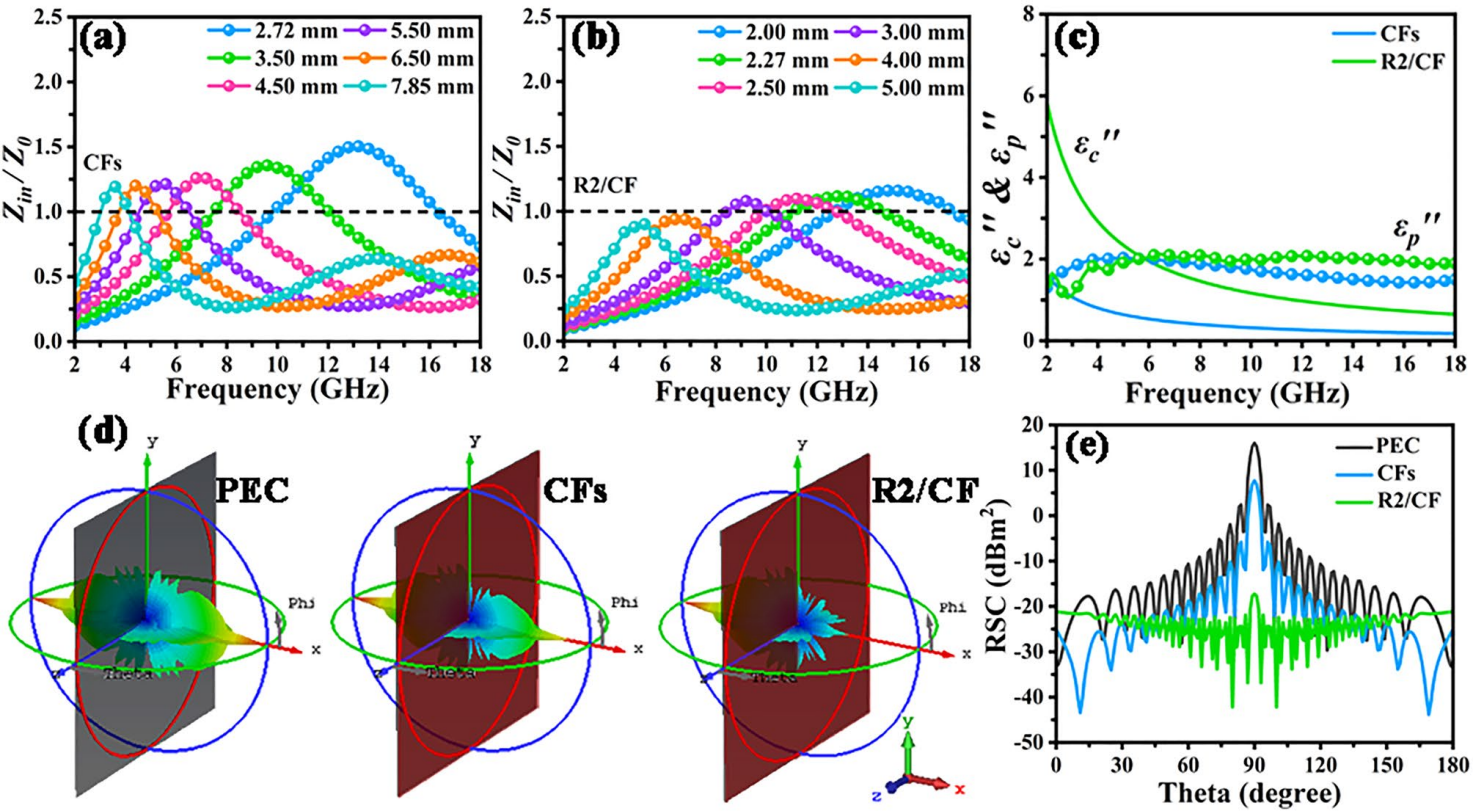
**a–c** Impedance-*f* curves, $\varepsilon^{\prime\prime}_{c}$, $\varepsilon^{\prime\prime}_{{\text{p}}}$ values for CFs and R2/CF, and **d, e** 3D RCS simulation and simulated RCS values at 0–180° incident angle for PEC, CFs and R2/CF

Generally speaking, optimal impedance matching characteristic implies more incident EMW permeating into the interior of absorber, which is instrumental for the subsequent EMW attenuation [49]. As shown in Fig. 7a, b, taking R2/CF with the filling ratios of 25 wt% as example, the comparison ${{Z_{in} } \mathord{\left/ {\vphantom {{Z_{in} } {Z_{0} }}} \right. \kern-0pt} {Z_{0} }}$ values indicate the designed 2D/3D RGO/CFs vdWs heterostructures achieve the much better impedance matching characteristic than the initial CFs, implying that the RGO addition improves the impedance matching characteristics. Additionally, the $\varepsilon^{\prime\prime}_{{\text{p}}}$ and $\varepsilon^{\prime\prime}_{c}$ values were achieved on basis of equations (Eq. S4) and (Eq. S5) in Supporting Information to evaluate the polarization and conduction loss capabilities, respectively [50, 51]. To determine the conductive loss based on Eq. S7, the $\sigma_{ac}$ values of CFs and R2/CF absorbers were acquired based on a Hall-effect system and are given in Table S2. It is apparent that the CFs exhibit the smaller value of $\varepsilon^{\prime\prime}_{c}$ than $\varepsilon^{\prime\prime}_{{\text{p}}}$ (Fig. 7c), implying the dominated contribution of polarization loss. Whereas, the achieved $\varepsilon^{\prime\prime}_{c}$ and $\varepsilon^{\prime\prime}_{{\text{p}}}$ values for R2/CF sample point to the major role of conduction loss at low frequency (below ca. 6.0 GHz) and polarization loss within 6.0–18.0 GHz frequency range. Moreover, R2/CF presents a significant enhancement in the $\varepsilon^{\prime\prime}_{c}$ and $\varepsilon^{\prime\prime}_{{\text{p}}}$ values compared to CFs in the whole tested frequency, indicating its apparently improved polarization and conduction loss capacities. In order to further verify the polarization relaxation loss, the Cole–Cole curves of CFs and RGO/CFs were drawn on the basic of Debye relaxation theory and are displayed in Fig. S8 [52]. Generally, a semicircle and long straight line corresponds to a Debye relaxation process and conduction loss, respectively [53]. Obviously, compared with CFs, RGO/CFs display relatively more semicircles in addition to linear regions, suggesting the enhancement of polarization loss in RGO/CFs. The obtained outcomes demonstrate that incorporating RGO to construct the 2D/3D RGO/CFs vdWs heterostructures simultaneously improves the impedance matching characteristic, conduction and polarization loss abilities. The linkage effect leads to their boosted EMW absorption properties. Besides, the radar cross section (RCS) measurement was carried out employing computer simulation technology (CST). As shown in Fig. 7d, the CST simulation outcomes reveal that the plate of perfect conductive layer (PEC) displays the strongest scattering signal. Whereas the PEC coated by CFs present the much higher signal intensity than that covered by R2/CF (2.5 mm thick). These contrast results further prove that most of EMW energy is effectively attenuated by 2D/3D RGO/CFs vdWs heterostructures. As compared in Fig. 7e, the obtained R2/CF sample exhibits the lowest RCS values (less than − 10 dB m^2^) within 0–180° angle region than PEC and CFs, which corresponds well with the prominent EMW absorption properties. On the other hand, R2/CF exhibits significant radar stealth property in the practical applications compared with PEC and CFs. The comparison of the designed RGO/CFs with the other recently reported carbon-based absorbers is detailed in Table S3. Overall, the resulting RGO/CFs exhibit outstanding performances, incorporating the characteristic of “strong,” “broad,” “thin” and “light.”

**Fig. 8:**
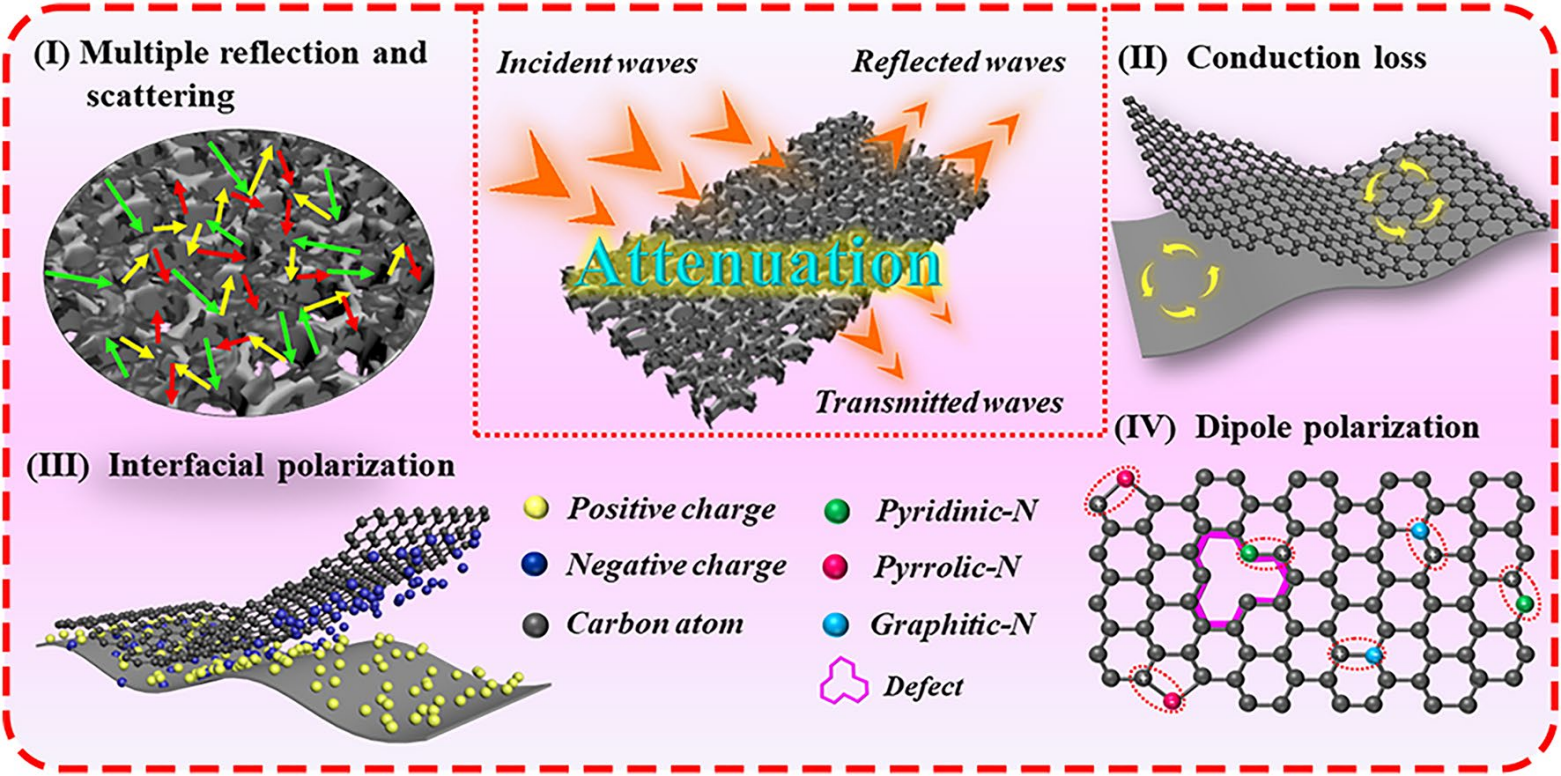
Diagrammatic sketch of EMW attenuation avenues for 2D/3D RGO/CFs vdWs heterostructures

Combined with the experiments and analyses demonstrated previously, it can be concluded that the designed cellular porous foams endow the absorber lightweight property and outstanding EMW absorption performances. For a more intuitive understanding, Fig. 8 summarizes the conceivable EMW attenuation mechanisms of 2D/3D RGO/CFs vdWs heterostructures. As a prerequisite, their typical mixed-dimensional cellular porous materials greatly correct impedance mismatch characteristics compared to single-dimensional structure. Based on the optimized impedance matching, most incident EMW can effectively permeate into the designed RGO/CFs absorbers and induced multiple reflection and scattering to achieve energy attenuation [54]. Meanwhile, the 2D RGO nanosheets and 3D CFs are cross-linked with each other to construct the wonderful conductive network. Benefiting from electron migration and hopping along among graphite nanocrystals, the conduction loss efficiently facilitates energy transformation from EMW energy to thermal energy, thus achieving attenuation [55, 56]. Besides the conduction loss, polarization loss is another crucial factor in accelerating EMW attenuation. Therein, the foam-like 2D/3D vdWs heterostructures and composite components provide numerous heterogeneous interfaces such as solid–air interfaces, different components interfaces, where interfacial polarization loss occurs when the different electrical properties charges accumulate on the heterogeneous interfaces [57, 58]. Another one, the dipole polarization loss deriving from defects, heteroatoms dopant as well as remaining polar groups inside RGO/CFs vdWs heterostructures also contribute to the attenuation of penetrated EMW [59, 60]. Overall, these special 2D/3D vdWs heterostructures consumedly optimize the impedance matching property and promote the dielectric loss ability, which contribute to their excellent EMW absorption performances.

### Versatility and Possible Application Prospects

**Fig. 9:**
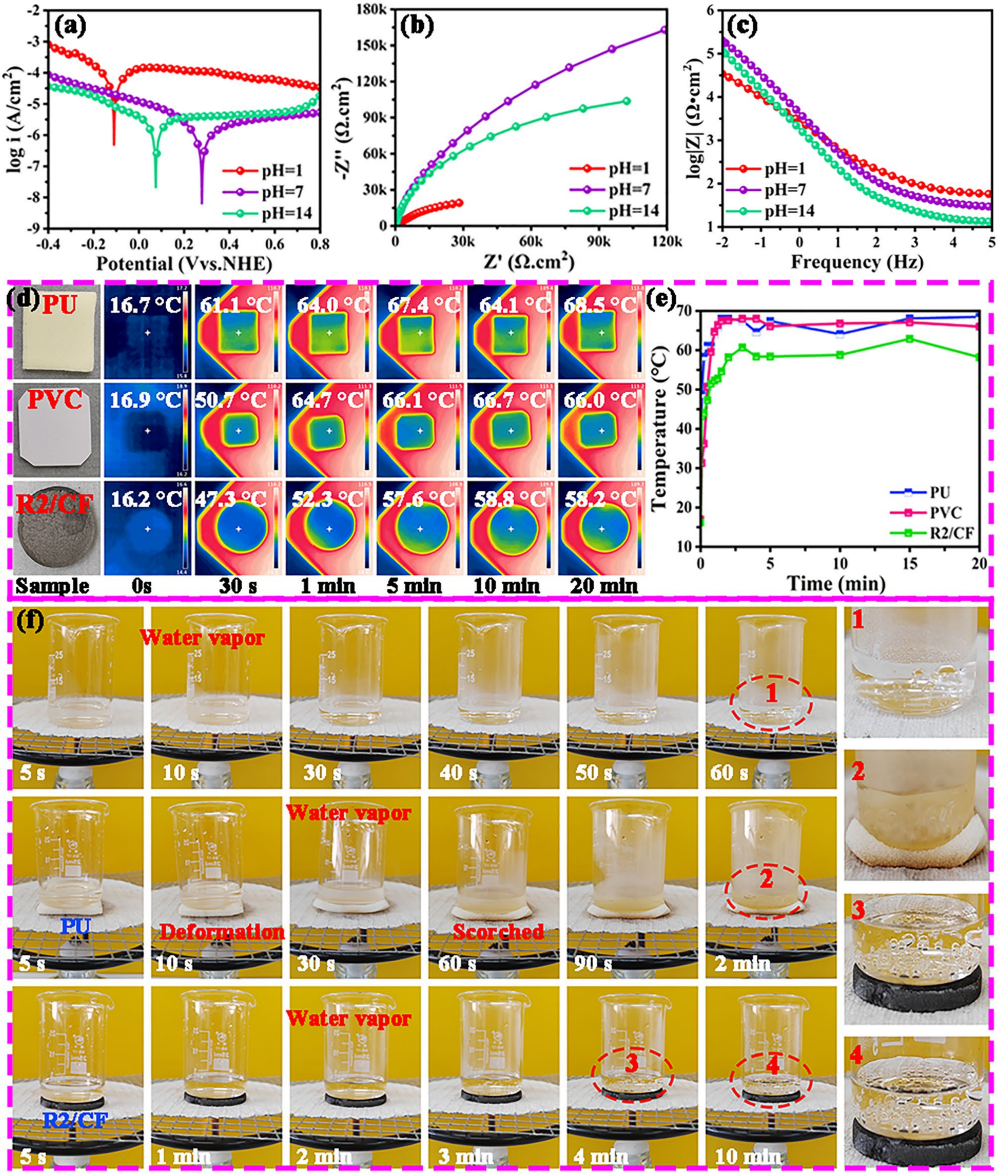
**a** Tafel curves, **b** EIS plots,** c** Bode plots of R2/CF in HCl solution (pH = 1), 3.5 wt% NaCl solution and KOH solution. **d, e** Thermal infrared images and corresponding temperature–time curves for PU, PVC, R2/CF captured at different times (from 5 to 20 min), and **f** Beaker containing 5 mL water placed on asbestos mesh, PU and R2/CF for heating by a spirit lamp

To investigate the practical application, we also conducted the corrosion resistance measurement using electrochemical measurement technique to further clarify the stability of designed RGO/CFs vdWs heterostructures in the various extreme conditions. In a typical experimental procedure, the obtained R2/CF sample was immersed in KOH solution (pH = 14), 3.5 wt% NaCl solution and HCl solution (pH = 1) for 30 min, respectively. As we all known, the high positive *E*_corr_ and low *I*_corr_ value imply the excellent corrosion resistance of sample [61]. From Tafel curves shown in Fig. 9a, compared to HCl (− 0.11 V and 158.9 μA) solution, the R2/CF sample exhibits a high positive *E*_corr_ and small *I*_corr_ values in the NaCl (0.278 V and 2.267 μA) and KOH (0.075 V and 8.201 μA) solutions, implying its better corrosion resistance under the neutral and alkaline conditions. Additionally, electrochemical impedance spectroscopy (EIS) measurement results (as presented in Fig. 9b) show that the R2/CF sample displays much larger radius of impedance arc under the NaCl and KOH solutions than HCl solution, indicating the strong charge transfer resistance ability and good anti-corrosion performance. As shown in Fig. 9c, it is once confirmed that the obtained sample has excellent corrosion resistance in neutral and alkaline condition. Based on the above findings, the outstanding anti-corrosion performance should be attributed to the high physical/chemical stability of carbon materials, dense heterostructures and excellent hydrophobicity. And strong hydrophobicity of R2/CF (water contact angle up to ca. 130° shown from Fig. S9) avoids the penetration of corrosion medium. Besides, good thermal insulating performance also protects microwave coating layers from high-temperature damage [62]. Accordingly, we provided intuitive comparison of insulation properties among R2/CF, commercial polyurethane (PU) foam and polyvinyl chloride (PVC) plate insulations. Notably, all of materials were set as 3.0 mm thick and the heating temperature was 100 °C. Figure 9d presents the thermal infrared photos of samples collected at various time points ranging from 0 s to 20 min. Visually, the detected temperatures of PU and PVC are stabilized at ca. 66 °C, whereas R2/CF remains at ca. 58 °C even 20 min, which profits from the highly porous heterostructure [63]. As compared in Fig. 9e, the thermal radiation performance of R2/CF is comparable to or even better than that of commercial material, implying the promising prospect of our designed RGO/CFs vdWs heterostructures in the practical applications. This satisfactory property is ascribed to the high porosity of 2D/3D R2/CF heterostructures, which extends the path of thermal transfer and further weakens the intensity of heat conduction and thermal radiation. More intuitively, the thermal insulation performance of R2/CF can be observed by heating a beaker containing 5 mL water without and with interlayer using a spirit lamp. As can be seen from Fig. 9f, after laying the beaker on asbestos mesh, water vapor begins to appear within 10 s and the water starts to boil at 60 s. After that, PU with 3.0 mm thick is selected as the control spacer, which is placed between the asbestos wire gauze and beaker. With the blocking effect of PU, steam emergence time and boiling time are extended to 30 s and 2 min, respectively. However, it can be seen that the PU deforms at 10 s and occurs apparently coking at 30 s. Finally, the same experiment was carried out using R2/CF as spacer. Amazingly, one can find from the enlarged images (named 3 and 4) that numerous minute bubbles have generated at the base of beaker at 4 min, it still fails to boil even after 10 min. It is evident that the R2/CF displays the much better thermal stability than the commercial PU. In general, these favorable outcomes indicate that the fabricated R2/CF owns the protruding thermal resistance performance and is suitable for aviation and space sectors and more complex environments.

## Conclusions

In summary, multifunctional 2D/3D RGO/CFs vdWs heterostructures could be meticulously engineered and synthesized via an oversimplified freeze-drying, immersing absorption, secondary freeze-drying and subsequent carbonization processes. The acquired outcomes indicated that the RGO introduction greatly optimized the impedance matching characteristics of 2D/3D RGO/CFs vdWs heterostructures and improved their polarization and conduction loss capabilities. And the EM parameters of 2D/3D RGO/CFs vdWs heterostructures could be effectively modulated by regulating the RGO content and carbonization temperature. Hence, the linkage effect of the optimized impedance matching and the enhanced dielectric loss capabilities endowed the designed 2D/3D RGO/CFs vdWs heterostructures with the excellent EMW absorption properties. As a result, the R2/CF displayed a low *RL*_min_ (− 50.58 dB) and broad EAB values (6.2 GHz). More importantly, the reasonable components design and mix-dimensional vdWs heterostructures contributed to significant radar stealth properties, good corrosion resistance performances as well as outstanding thermal insulation capabilities of 2D/3D RGO/CFs vdWs heterostructures, displaying more great potential in complex and variable conditions.

## Supplementary Information

Below is the link to the electronic supplementary material.

Supplementary file1 (DOCX 1802 KB)
