# Supplementary material for: Mixed-Dimensional Assembly Strategy to Construct Reduced Graphene Oxide/Carbon Foams Heterostructures for Microwave Absorption, Anti-Corrosion and Thermal Insulation
Source: Nanomicro Lett. 2024 Jun 17;16:221. doi: 10.1007/s40820-024-01447-9 (PMC11183034; doi:10.1007/s40820-024-01447-9)
Supplement: Supplementary file 1 — Supplementary file1 (DOCX 1802 KB) [file 40820_2024_1447_MOESM1_ESM.docx]

Supporting Information for

**M****ixed-Dimensional Assembly Strategy to Construct Reduced Graphene Oxide/Carbon Foams Heterostructures for Microwave Absorption, Anti-corrosion and Thermal Insulation**

Beibei Zhan^1^, Yunpeng Qu^1^, Xiaosi Qi^1^,*, Junfei Ding^1^, Jiao-jing Shao^2^, Xiu Gong^1^, Jing-Liang Yang^1^, Yanli Chen^1^, Qiong Peng^1^, Wei Zhong^3^, Hualiang Lv^4^,*

^1^College of Physics, Guizhou Province Key Laboratory for Photoelectrics Technology and Application, Guizhou University, Guiyang City 550025, P. R. China

^2^College of Materials and Merallurgy, Guizhou University, Guiyang City 550025, P. R. China

^3^National Laboratory of Solid State Microstructures and Jiangsu Provincial Laboratory for NanoTechnology, Nanjing University, Nanjing 210093, P. R. China

^4^Department of Materials Science and Laboratory of Advanced Materials, Fudan University, Shanghai, 200433, P. R. China

*Corresponding authors. E-mail: [xsqi@gzu.edu.cn](mailto:xsqi@gzu.edu.cn) (Xiaosi Qi), [lv_hl@fudan.edu.cn](mailto:lv_hl@fudan.edu.cn) (Hualiang Lv)

**Supplementary Equations, Tables and Figures**

**Equations:**

$Z_{in}=Z_{0}\sqrt{\frac{\mu}{\varepsilon}}\tanh\left(j\frac{2\pi fd\sqrt{\mu\varepsilon}}{c}\right)$ (Eq. S1)

$RL=20\log\left|\frac{Z_{in}-Z_{0}}{Z_{in}+Z_{0}}\right|$ (Eq. S2) $\alpha=\frac{\sqrt{2}\pi f}{c}\sqrt{\left(\mu''\varepsilon''-\mu'\varepsilon'\right)+\sqrt{\left(\mu''\varepsilon''-\mu'\varepsilon'\right)^{2}+\left(\varepsilon'\mu''+\varepsilon''\mu'\right)^{2}}}$ (Eq. S3)

$\varepsilon'=\varepsilon_{\infty}+\frac{\varepsilon_{s}-\varepsilon_{\infty}}{1+\omega^{2}\tau^{2}}$ (Eq. S4)$\varepsilon''=\frac{\varepsilon_{s}-\varepsilon_{\infty}}{1+\omega^{2}\tau^{2}}\omega\tau\text{+}\frac{\sigma_{ac}}{\omega\varepsilon_{0}}\text{=}\varepsilon''_{\text{p}}+\varepsilon''_{c}$ (Eq. S5)$\left(\varepsilon'\text{-}\frac{\varepsilon_{s}+\varepsilon_{\infty}}{2}\right)^{2}\text{+}\left(\varepsilon''\right)^{2}\text{=}\left(\frac{\varepsilon_{s}-\varepsilon_{\infty}}{2}\right)^{2}$ (Eq. S6)

$\varepsilon''_{c}=\frac{\sigma_{ac}}{\omega\varepsilon_{0}}$ (Eq. S7)

where *d*, *f*, *c*, $\varepsilon$, $\mu$, *Z_0_*, *Z_in_*, $\varepsilon_{\infty}$, $\varepsilon_{s}$, $\varepsilon_{0}$, $\omega$, $\sigma_{ac}$, $\tau$ and $\alpha$ represent the thickness of sample, frequency of EMW, velocity of light, complex permittivity, complex permeability, impedance of air, characteristic impedance of material, relative dielectric permittivity at the high frequency limit, static permittivity, the vacuum dielectric constant, angular frequency, conductivity, relaxation time and attenuation constant, respectively.

**Table S1** EM parameters and performances for R2/CF-600 and R2/CF-700 with different filling ratios of 15 wt%, 20 wt% and 25 wt%

| EM parameters &  Performances  Samples | $\varepsilon'$ | $\varepsilon''$ | RL_min_**/**dB  *d_m_***/**mm | EAB/GHz  *d_m_***/**mm |
| --- | --- | --- | --- | --- |
| R2/CF-600-15 wt% | 5.420-3.519 | 2.123-0.948 | -33.06  7.35 | 4.8  7.47 |
| R2/CF-600-20 wt% | 6.516-3.966 | 2.812-1.148 | -20.29  9.76 | 5.2  7.15 |
| R2/CF-600-25 wt% | 8.466-4.463 | 4.642-1.729 | -65.67  4.92 | 5.4  2.48 |
| R2/CF-700-15 wt% | 11.904-7.321 | 5.987-3.142 | -52.05  3.32 | 5.0  1.85 |
| R2/CF-700-20 wt% | 19.347-10.318 | 13.453-6.037 | -14.80  1.47 | 4.2  1.47 |
| R2/CF-700-25 wt% | 29.040-12.758 | 29.296-9.797 | -8.87  1.12 | 0.0  0.00 |

**Table S2** Alternative conductivity for CFs and R2/CF

| Samples | CFs | R2/CF |
| --- | --- | --- |
| $\sigma_{ac}\left(S/m\right)$ | 0.17877225492878 | 0.65069017381954 |

**Table S3** Contrastive table about performances of designed RGO/CFs with the recently reported representative carbon-based absorbers

| Sample | RL_min_ (dB ) | *dm* (mm) | EAB (GHz) | *dm* (mm) | filling ratio (wt%) | References |
| --- | --- | --- | --- | --- | --- | --- |
| α-Fe_2_O_3_@CMTs | -84.01 | 3.19 | 7.17 | 2.65 | 50 | [S1] |
| NbS_2_/rGO | -55.00 | 2.30 | 6.40 | 2.60 | 40 | [S2] |
| MXene/MWCNTs@C-Co | -70.70 | 2.04 | 5.67 | 2.04 | 15 | [S3] |
| Fe_3_O_4_-Fe@CNFs/Al-Fe_3_O_4_-Fe | -59.30 | 4.30 | 5.60 | 2.20 | 30 | [S4] |
| N-doped hollow carbon spheres@NiO/Ni | -44.04 | 2.00 | 4.38 | 1.70 | 12 | [S5] |
| hollow SiC/C | -60.80 | 2.70 | 5.10 | 1.80 | 35 | [S6] |
| FCMT/CuCo_2_S_4_/PAN | -56.61 | 1.75 | 5.54 | 1.75 | 50 | [S7] |
| R4/CF | -27.81 | 1.73 | 6.00 | 2.04 | 25 | This work |
| R2/CF | -50.58 | 2.50 | 6.20 | 2.27 | 25 | This work |


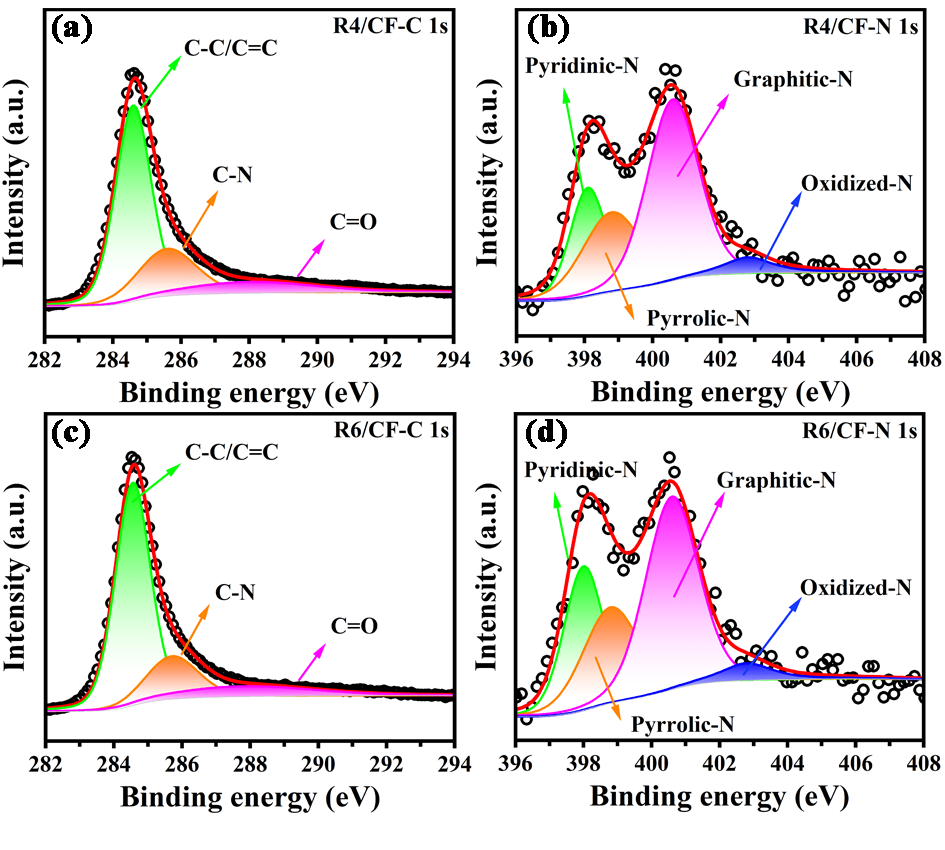


**Fig. S1** High resolution XPS spectra of C 1s and N 1s for **a, b** R4/CF and **c, d** R6/CF


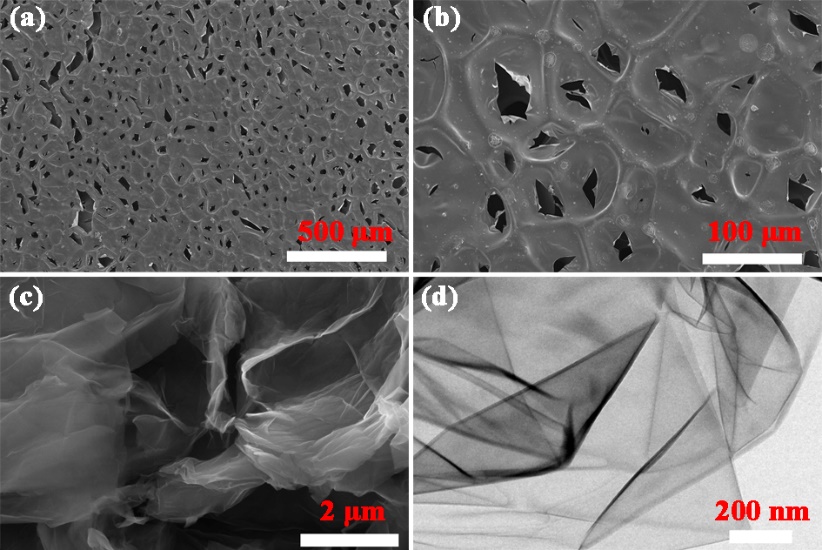


**Fig. S2 a, b** SEM images of CGFs, **c** SEM and **d** TEM images of GO





**Fig. S3** EAB and d_m_ curves for CFs with a 25 wt% filling ratio


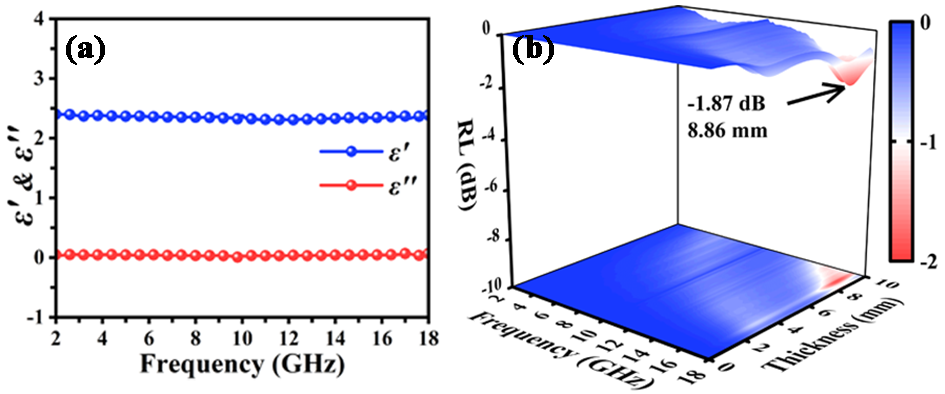


**Fig. S4 a** EM parameter and **b** EMW absorption performance of GO with the filling ratio of 25 wt%





**Fig. S5** Attenuation constant curves of CFs and 2D/3D RGO/CFs at 25 wt% filling ratio


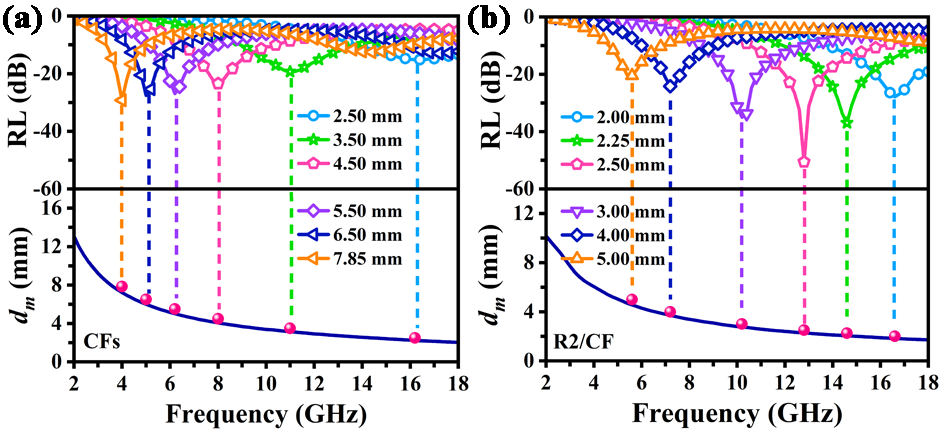


**Fig. S6** Comparison results between the experimental *d_m_* values and theoretical curves for **a** CFs and **b** R2/CF


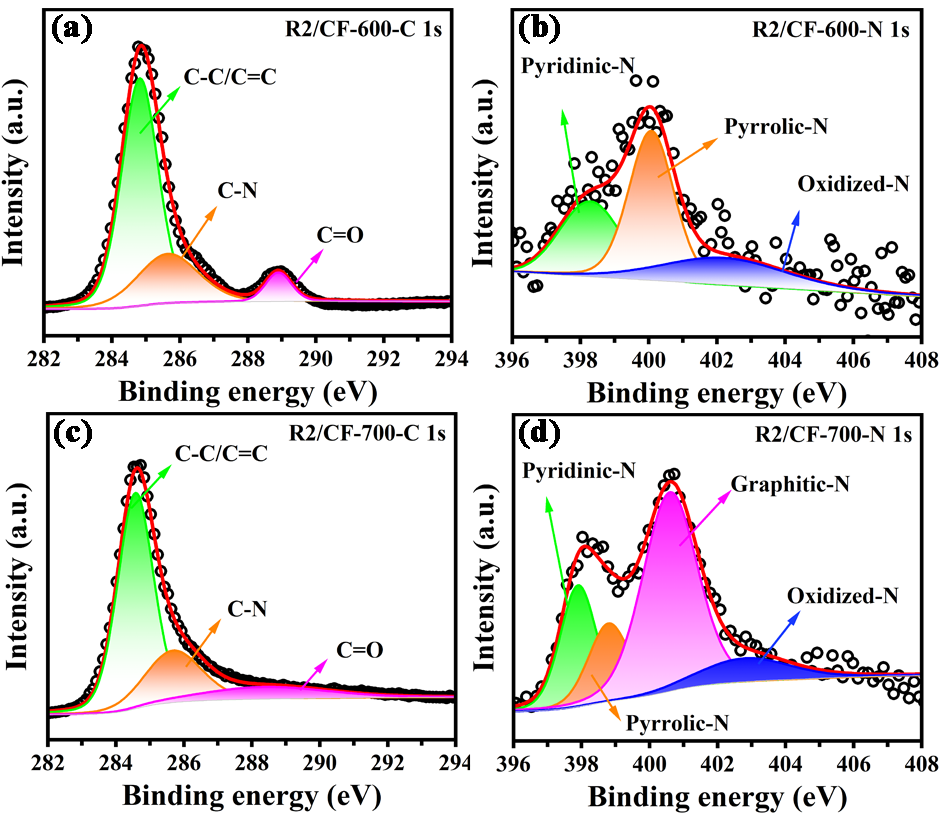


**Fig. S****7** High resolution XPS spectra of C 1s and N 1s for **a, b** R2/CF-600 and **c, d** R2/CF-700


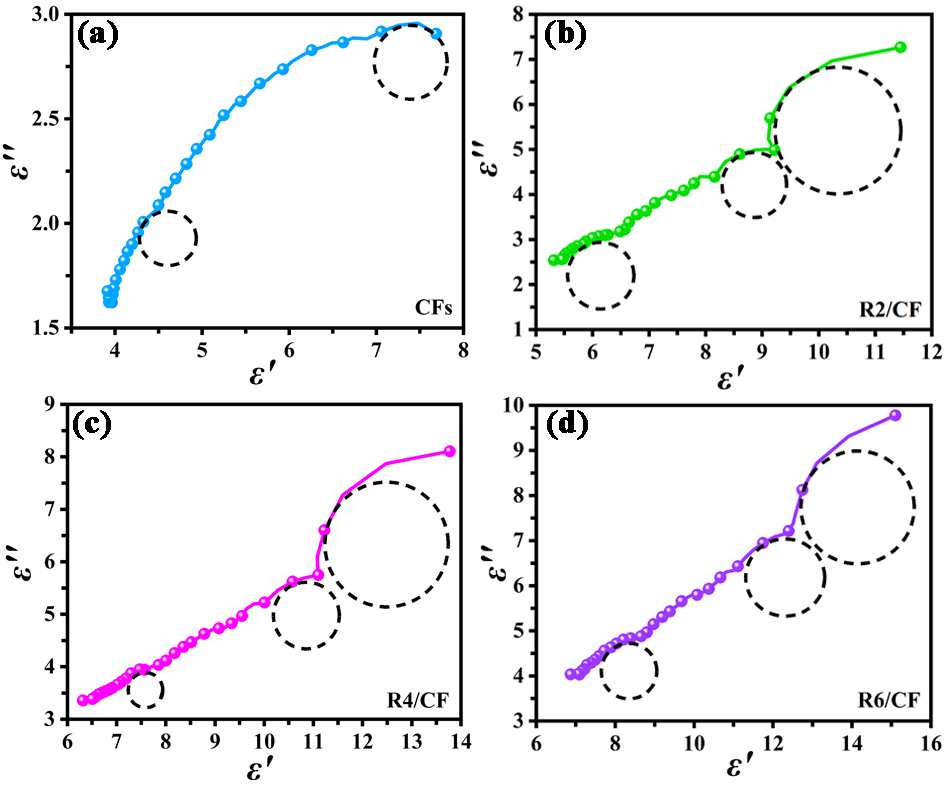


**Fig. S8** Cole-Cole curves of **a** CFs and **b-d** RGO/CFs with filling ratio of 25 wt%


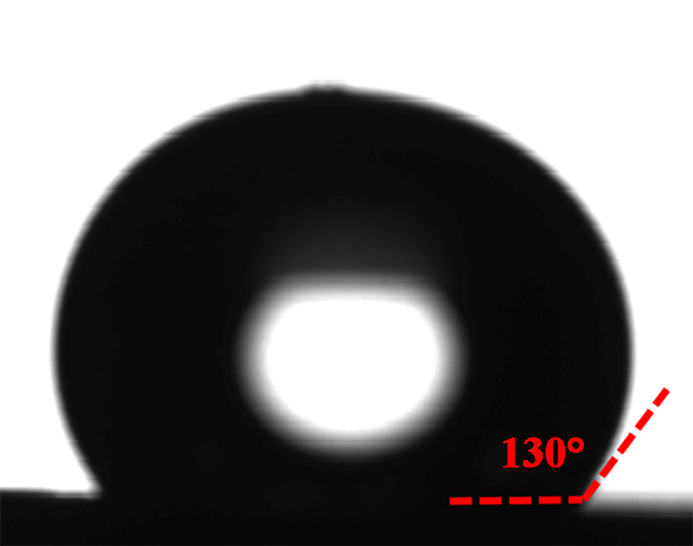


**Fig. S9** Water contact angle of R2/CF sample

**Supplementary References**

1. H. Ren, T. Zhu, L. Feng, Q. Wu, K. Wang et al., Atomic valence reversal-induced polarization resonance spurs highly efficient electromagnetic wave absorption in α-Fe_2_O_3_@carbon microtubes. Nano Lett. **24**(11), 3525-3531 (2024). <https://doi.org/10.1021/acs.nanolett.4c00532>
2. L. Yao, Y. Wang, J. Zhao, Y. Zhu, M. Cao, Multifunctional nanocrystalline‐assembled porous hierarchical material and device for integrating microwave absorption, electromagnetic interference shielding, and energy storage. Small **19**(25), 2208101 (2023). <https://doi.org/10.1002/smll.202208101>
3. Z. Wu, X. Tan, J. Wang, Y. Xing, P. Huang et al., MXene hollow spheres supported by a C–Co exoskeleton grow MWCNTs for efficient microwave absorption. Nano-Micro Lett. **16**, 107 (2024). <https://doi.org/10.1007/s40820-024-01326-3>
4. X. Liu, J. Zhou, Y. Xue, X. Lu, Structural engineering of hierarchical magnetic/carbon nanocomposites via in situ growth for high-efficient electromagnetic wave absorption. Nano-Micro Lett. **16**, 174 (2024). <https://doi.org/10.1007/s40820-024-01396-3>
5. B. Li, Z. Ma, X. Zhang, J. Xu, Y. Chen et al., NiO/Ni heterojunction on N‐doped hollow carbon sphere with balanced dielectric loss for efficient microwave absorption. Small **19**(12), 2207197 (2023). <https://doi.org/10.1002/smll.202207197>
6. L. Gai, Y. Wang, P. Wan, S. Yu, Y. Chen et al., Compositional and hollow engineering of silicon carbide/carbon microspheres as high-performance microwave absorbing materials with good environmental tolerance. Nano-Micro Lett. **16**, 167 (2024). <https://doi.org/10.1007/s40820-024-01369-6>
7. R. Peymanfar, E. Selseleh-Zakerin, A. Ahmadi, S.H. Tavassoli, Architecting functionalized carbon microtube/carrollite nanocomposite demonstrating significant microwave characteristics. Sci. Rep. **11**(1), 11932 (2021). <https://doi.org/10.1038/s41598-021-91370-5>
